# Supplementary material for: Private sector tuberculosis care quality during the COVID-19 pandemic: a repeated cross-sectional standardised patients study of adherence to national TB guidelines in urban Nigeria
Source: BMJ Glob Health. 2024 Nov 14;9(11):e015474. doi: 10.1136/bmjgh-2024-015474 (PMC11575386; doi:10.1136/bmjgh-2024-015474)
Supplement: online supplemental file 2 [file bmjgh-9-11-s002.pdf]

Supplement 2: Reflexivity Statement

1. *How does this study address local research and policy priorities?*

Nigeria is one of eight countries that account for more than two-thirds of new tuberculosis (TB) cases each year. Prior to this study, the SHOPS Plus project implemented a standardized patients (SP) study on the quality of TB care among the facilities supported through a public-private mix (PPM) approach to improve private sector TB case detection and treatment. This study aligns with the goals of the Federal Ministry of Health's TB PPM Action Plan, and addresses priority research questions for other similar LMICs surrounding the impact of COVID-19 on quality of TB care and its implications for future PPM efforts.

2. *How were local researchers involved in study design?*

The research study was co-designed by the co-PIs (MP, JD) with assistance from EB, BOF, LR, and COO, and in partnership with others from the Federal Ministry of Health (CA, OCA) and from the SHOPS Plus project team based in Nigeria.

3. *How has funding been used to support the local research team?*

The local research team based in Nigeria was funded to conduct the SP research. Expenses included time and materials for the SPs, staff time for research study support, and expenses for disseminating results to local stakeholders.

4. *How are research staff who conducted data collection acknowledged?*

The research staff who supervised data collection (LR, BOF, MT) are included as co-authors. The SPs who conducted the visits are thanked in the acknowledgments section of this manuscript.

5. *Do all members of the research partnership have access to study data?*

All members of the partnership have access to all study data.

6. *How was data used to develop analytical skills within the partnership?*

Analyses for this manuscript formed part of the MSc training of the first author (AS).

7. *How have research partners collaborated in interpreting study data?*

Research partners were integral to the process of interpreting and contextualizing the statistical findings based on their extensive knowledge of the local health system in Nigeria, particularly BOF, LR, EB, MT, CA, and OCA. Their contributions were added to by the other co-authors and senior authors, COO, MP, and JD, who contributed additional expertise on the global context of TB and COVID-19.

8. *How were research partners supported to develop writing skills?*

Research partners were not directly supported to develop writing skills through this work.

9. *How will research products be shared to address local needs?*

Results of this study were disseminated to local stakeholders in Nigeria and international stakeholders in 2021 (https://shopsplusproject.org/resource-center/webinar-assessing-quality-tb-services-nigeria-results-mystery-client-survey).

10. How is the leadership, contribution and ownership of this work by LMIC researchers recognised within the authorship?

Two authors (CA and OCA) are representatives of the Federal Ministry of Health in Nigeria, who authorized the study and who have oversight of the data collected in this study. Two additional authors (BOF and MT) are members of the SHOPS Plus team based in Nigeria who have contributed extensively to the work reflected in this manuscript. Finally, the last author is a native Nigerian now based at a Canadian academic institution (COO).

11. How have early career researchers across the partnership been included within the authorship team?

Eight early career researchers have been incorporated as authors (AS, LR, MT, MAHK, LH, NAV, BD, COO). The last author is also an early career researcher from an LMIC country of origin.

12. How has gender balance been addressed within the authorship?

Five authors are male (BJ, MAHK, BD, JD, MP) and eight authors are female (AS, EB, LR, BOF, MT, LH, NAV, COO).

13. How has the project contributed to training of LMIC researchers?

This project has not directly contributed to training of LMIC researchers.

14. How has the project contributed to improvements in local infrastructure?

This project has not directly contributed to improvements in local infrastructure.

15. What safeguarding procedures were used to protect local study participants and researchers?

All staff contracted to serve as SPs and supervisors underwent a six-day training including extensive discussions and role plays. During the training, SPs conducted several rounds of full mock interviews to help them immerse in their roles and avoid pitfalls that could compromise the study. The training also emphasized mitigation protocols and techniques to protect SPs from harm (i.e., avoiding invasive procedures such as blood draws, administration of medicines, etc.).
